# Genetic insights into acute lymphoblastic leukemia: the role of *MDR1* and *IL18* polymorphisms in Egyptian children

**DOI:** 10.1186/s12885-025-15132-6

**Published:** 2025-11-19

**Authors:** Ali Nabeel Mahdi, Afaf M. Elsaid, Maha Abdelmoneim Mohammed, Mai M. Madkour, A.F. Abdel-Aziz

**Affiliations:** 1 Biochemistry Division, Department of Chemistry, Faculty of Science, Mansoura University, Mansoura, 35516 Egypt; 2 Mansoura Children Hospital, Faculty of Medicine, Mansoura University, Mansoura , 35516 Egypt; 3 Hematology and Oncology unit, Faculty of Medicine, Mansoura University, Mansoura , 35516 Egypt

**Keywords:** Acute Lymphoblastic Leukemia, Multidrug Resistance Gene, Interleukin 18, Polymorphism

## Abstract

**Background:**

The most prevalent cancer in pediatric is acute lymphoblastic leukemia (ALL). The multidrug resistance gene (*MDR1*) encodes the membrane transport protein P-glycoprotein (P-gp), which acts as an efflux pump. Interleukin 18 (*IL18*), an 18-kilodalton cytokine, plays a complex role in cancer, exhibiting both anti-cancer and pro-cancer properties. This study aims to investigate the association between polymorphisms in the *MDR1* gene (G2677T, rs2032582) and *IL18* gene variants (607C > A, rs1946518 and − 137G > C, rs187238) and their potential role in susceptibility to pediatric ALL in an Egyptian population. We hypothesize that specific polymorphisms in *MDR1* and *IL18* genes are significantly associated with an increased risk of developing pediatric ALL, and that these genetic variants may serve as potential biomarkers for early detection and prognosis.

**Methods:**

*MDR1* (G2677T) rs2032582, *IL18* (607C > A) rs1946518, and *IL18* (-137G > C) rs187238 variants were genotyped in 100 childhood ALL (58 male and 42 female) cases and 100 healthy controls (49 male and 51 female) using the tetra-primer amplification refractory mutation system-polymerase chain reaction (T-ARMS-PCR) technique.

**Results:**

The statistical analysis of the results indicated that the *MDR1* (G2677T) rs2032582 genotypes (*p* = 0.051) and allele distribution (*p* = 0.217) showed no discernible variations between the controls and cases. The data indicate a strong correlation between the TT genotype and an elevated risk of ALL in both sexes. The allele frequency and genotype of *IL18* (607C > A) rs1946518 exhibited a significant difference (*p* = 0.001) between the controls and cases. The results indicated a substantial difference in allele frequency (*p* = 0.0006) and genotype of the *IL18* (-137G > C) polymorphism (*p* = 0.001) between the controls and cases.

**Conclusions:**

The results suggest that the *MDR1* (G2677T) rs2032582 polymorphism may not serve as a dependable prognostic indicator of the disease. In contrast, *IL18* (607C > A) rs1946518 and *IL18* (-137G > C) rs187238 polymorphisms may affect susceptibility to pediatric leukemia, indicating that *IL18* could be a possible biomarker for the early identification of ALL.

**Supplementary Information:**

The online version contains supplementary material available at 10.1186/s12885-025-15132-6.

## Background

Acute lymphoblastic leukemia (ALL) is a particularly aggressive form of hematopoietic cancer that is both physiologically diverse and clinically aggressive. The clonal proliferation and accumulation of immature lymphoid progenitor cells are the causes of this condition, which ultimately results in extensive infiltration of the thymus, peripheral blood, bone marrow, and lymphoid organs [1, 2]. It represents the most common pediatric malignancy, accounting for approximately 80% of all acute leukemia cases in children [3]. ALL can arise from distinct lymphoid progenitor lineages, most commonly giving rise to B-cell or T-cell leukemia, and less frequently to mixed-lineage leukemias. The pathogenesis of ALL is frequently driven by aberrant gene expression, commonly resulting from chromosomal translocations that disrupt normal hematopoietic development. About 20% of pediatric cancers in Egypt are ALL, with an estimated yearly incidence of 4 cases per 100,000 pediatric [4]. Although the precise etiology of ALL remains unclear, pediatric cases have been associated with various risk factors, including environmental exposures, ionizing radiation, inherited genetic syndromes, and underlying genetic susceptibility [5].

The multidrug resistance gene (*MDR1*), also known as ATP-binding cassette sub-family B member 1, is located on chromosome 7 at the position 21.1 [6]. Its complementary DNA measures approximately 4.5 kilobases in length. This gene features a core promoter region and consists of 28 exons [7]. The *MDR1* gene is responsible for the encoding of P-glycoprotein (P-gp), which is a membrane transport protein that performs the function of an efflux pump. This protein is also required for the transport of various chemicals, including several drugs, through cell membranes in both directions. P-gp is primarily expressed in the epithelial cells of the gut, where it plays a crucial role in the absorption, distribution, and the overall pharmacological effects of many medications [8]. The prevalent *MDR1* mutation in the coding region is the mutation of G2677T. This polymorphism has been significantly correlated with variability of plasma levels of P-gp substrates following increasing or decreasing trend [9]. It is a significant problem that the *MDR1* (G2677T) gene polymorphism constitutes a barrier to successful treatment of cancer patients treated by chemotherapy including leukemia patients [10]. A number of studies have evaluated the relationship between the *MDR1* (G2677T) polymorphism and leukemia risk; however, the results have been inconsistent [11].

Interleukin 18 (*IL18*) is located on chromosome 11q22.2-q22.23 and consists of six exons. This cytokine plays a crucial role in the inflammatory response, and chronic inflammation is closely related with the development of several forms of cancer as well as their progression [12, 13]. *IL18* is a pleiotropic pro-inflammatory cytokine and a member of the *IL1* superfamily, known as an interferon-gamma-inducing factor. *IL18* is described as a double-edged sword, exhibiting both anti-tumor and pro-tumor effects [14]. On the anti-tumor side, *IL18* activates natural killer cells and promotes Th1 responses. It also stimulates the secretion of various cytokines and chemokines, leading to the elimination of tumor cells, thus contributing to both innate and adaptive immunity [15].

Conversely, *IL18* can also stimulate various tumor cell behaviors, including angiogenesis, proliferation, migration, and immune evasion [16]. Elevated levels of *IL18* have been observed in various solid tumors, such as gastric, lung, and breast cancers [17, 18]. *IL18* expression levels are regulated by at least two polymorphisms: (607C > A) rs1946518 and (−137G > C) rs187238 [19]. The (−137G > C) rs187238 polymorphism in the *IL18* gene promoter may modify the binding site for the histone 4 transcription factor-1, leading to changes in *IL18* expression levels [20]. Additionally, while there is no evidence of *IL18* overexpression in childhood ALL patients, elevated *IL18* levels have been documented in various leukemia types, including ALL [21].

## Methods

### Study participants

This study included 100 pediatric patients diagnosed with ALL (58 males and 42 females). As a control group, 100 unrelated, healthy blood donors (51 females and 49 males) without any history of chronic illness, matched to the case group by geographic location and ethnic background, were selected. Pediatric ALL patients were evaluated by the Pediatric Oncology Department at the Oncology Centre, Mansoura University, Egypt, between July 2024 and April 2025 while undergoing treatment or routine follow-ups.

The criteria for the study included pediatric patients under 18 years of age who were newly diagnosed and had no family history of leukemia or other cancers. The selection criteria were applied to individuals presenting with any concurrent acute or chronic inflammatory disorders, cardiovascular diseases, or other malignancies. Participants were mandated to exhibit no indications of personal or familial cancer history or other chronic diseases. Individuals over the age of 18, as well as those with acute or chronic diseases that could hinder their participation in the experiment, were also excluded, along with patients diagnosed with other oncological diseases. Informed consent for enrollment in the study was secured from the legal guardians of all participants. Supportive information and demographic data were collected from all participants or their parents.

## Sample collection

A total of 5 mL of peripheral blood samples were collected from 100 patients diagnosed with ALL, utilizing sterile and single-use plastic syringes. Additionally, 100 control samples were obtained from a healthy cohort, divided into 2 mL of blood collected in EDTA tubes for DNA extraction and hematology investigation. The extraction was conducted in accordance with the standard protocols specified in the QIAGEN QIAamp DNA Blood Mini Kit (QIAGEN, Germany), adhering to the manufacturer’s guidelines [22].

Patients had a comprehensive medical history review along with general and localized clinical examinations. The clinical data for ALL patients were retrieved from their archives. Such as cases and control groups as regards the following laboratory findings; Age, gender, Total Leukocyte Count (TLC), Hemoglobin (HB), Glutamate Pyruvate Transaminase (GPT), Glutamate Oxaloacetate Transaminase (GOT), Creatinine, Bilirubin and Albumin.

### Amplification of the MDR1 (G2677T) rs2032582, of IL18 (607C > A) rs1946518 and IL18 (−137G > C) rs187238 variants by T-ARMS-PCR method

The *MDR1* (G2677T) rs2032582, *IL18* (607C > A) rs1946518, and *IL18* (−137G > C) rs187238 variants were analyzed using the tetra-primer amplification refractory mutation system-polymerase chain reaction (T-ARMS-PCR) method. The PCR reaction mixture was prepared in a final volume of 25 µl, consisting of 4 µl of template DNA, 2 µl of each primer, and 13 µl of Taq master mix (Thermo Scientific). Amplification was performed using an Applied Biosystems™ Thermal Cycler. Subsequently, the resulting PCR products were separated by electrophoresis on a 2% agarose gel containing 0.5 µg/ml ethidium bromide and visualized under UV light through transillumination. Genotyping images were captured using a digital camera. Each polymorphism was amplified using its specific primers and PCR conditions as detailed in Table 1.

**Table 1:** Primers and PCR product for each gene polymorphisms

SNP (Gene)	Sequence (5’ → 3’)	PCR Program	PCR Product Length
*MDR1* (G2677T) rs2032582	Forward outer primer 5’ TCAGAAAATAGAAGCATGAGTTGTGA-3’ [23]	Initial denaturation: 94 °C, 5 min Denaturation: 95 °C, 2 min Annealing: 56 °C, 1 min Extension: 72 °C, 1 min Final extension: 72 °C, 10 min (30 cycles)	219 bp (G**-**allele) 290 bp (T**-**allele) 453 bp (internal control)
	Reverse outer primer 5’ GAACTGGCTTTGCTACTTTCTGTAAG-3’.		
	Forward inner primer 5’ CACTGAAAGATAAGAAAGAACTAGAAGATG- 3’		
	Reverse inner primer 5’ TATTTAGTTTGACTCACCTTCCCGGA- 3’		
*IL18* (607C > A) rs1946518	Forward outer 5′ - CCTACAATGTTACAACACTTAAAAT − 3′ [24, 25]	Initial denaturation: 95 °C, 5 min Denaturation: 95 °C, 30 s annealing: 53 °C, 20 s Extension: 72 °C, 30 s Final extension: 72 °C, 10 min (30 cycles)	208 bp (C allele) 278 bp (A allele) 440 bp (internal control)
	Reverse outer 5′ -ATAAGCCCTAAATATATGTATCCTTA − 3′		
	Forward inner 5′ -GATACCATCATTAGAATTTTGTG − 3′		
	Reverse inner 5′ -GCAGAAAGTGTAAAAATTATCAA − 3′		
*IL18* (−137G > C) rs187238	Forward outer 5′-AGATGCTTCTAATGGACTAAGGAG-3′ [26]	Initial denaturation: 94 °C, 3 min Denaturation: 94 °C, 20 s Annealing: 54 °C, 20 s Extension: 72 °C, 20 s Final extension: 72 °C, 5 min (40 cycles)	261 bp (C and G alleles) 446 bp (internal control)
	Reverse outer 5′-GGCAAAATGCACTGGGAGACAAT-3′,		
	Forward inner 5′-GCCCCAACTTTTACGGAAGAATAG-3′		
	Reverse inner 5′-ATGTAATATCACTATTTTCATGAACTG-3′.		

## Statistical analysis and data interpretation

Genotypic data for the *MDR1* (G2677T) rs2032582, *IL18* (− 607C > A) rs1946518, and *IL18* (− 137G > C) rs187238 polymorphisms were collected, structured, and statistically analyzed utilizing SPSS software, version 26 (IBM Corp., Armonk, NY, USA). Qualitative data were reported as frequencies and percentages, whilst quantitative data were conveyed as mean ± standard deviation for normally distributed variables and as median (minimum–maximum) for non-normally distributed variables. The Kolmogorov–Smirnov test was employed to evaluate normality. Using the chi-square test, Hardy-Weinberg equilibrium (HWE) was evaluated for every single nucleotide polymorphism (SNP) in the control group to determine whether observed genotype frequencies differed from expected distributions. SNPs that significantly deviated from HWE (*p* < 0.05) were excluded from subsequent analyses to minimize the potential influence of genotyping errors or population stratification.

Group comparisons for qualitative variables were conducted using the Chi-square test, Fisher’s exact test, or Monte Carlo simulation, as applicable. The Mann–Whitney U test was employed to compare two research groups about non-normally distributed quantitative data. The autonomous samples The Student’s t-test was employed to compare two groups for normally distributed quantitative data. A binary logistic regression analysis was performed to assess the cumulative impact of several independent variables on a binary outcome, employing stepwise, forward Wald, or enter approaches as suitable.

## Results

### Demographic, laboratory and clinical parameters between studied groups

The present study included 100 ALL cases and 100 individuals in the control group. A comparison of demographic characteristics between the groups showed no significant differences in age (*p* = 0.159) or gender (*p* = 0.202). The mean age of cases was 9.9 ± 4.1 years, compared to 9.1 ± 3.7 years in the control group. Regarding gender distribution, 58% of the ALL patients were male and 42% were female, while the control group consisted of 49% males and 51% females. When comparing laboratory results between the cases and control groups, there were significant differences in TLC, Hb, GPT, GOT, and creatinine levels (*p* < 0.001 for each). Higher values of TLC, Hb, and creatinine were observed in the control group compared to cases, while GPT and GOT levels were lower in the control group than in the ALL cases (Table 2).

**Table 2:** Comparison of demographic and laboratory parameters between studied groups

Parameters Mean ± SD or *N* (%)	Cases group (*N* = 100)	Controls group (*N* = 100)	*P* value
Age/years	9.9 ± 4.1	9.1 ± 3.7	0.159
Gender			
Male	58(58)	49(49)	0.202
Female	42(42)	51(51)	
Albumin	4.1 ± 0.5	4.1 ± 0.5	0.800
GPT	53.7(9–794)	28(20–38)	< 0.001*
GOT	33.5(10–561)	24(16–39)	< 0.001*
Creatinine	0.5(0.2–51.3)	0.9(0.8–1.1)	< 0.001*
Bilirubin	0.7(0.2–4.3)	0.7(0.4–0.9)	0.477

Used test: Student t test, Chi-Square test, Mann Whitney U test, *statistically significant, data expressed as mean ± SD, median (range)

*TLC* Total Leukocyte Count, *HB* Hemoglobin, *GPT* Glutamate Pyruvate Transaminase, *GOT* Glutamate Oxaloacetate Transaminase

The clinical presentation of the cases showed that 71% of patients had fever, 98% had pallor, 64% had bone pain, 72% had increased weight, 13% had arthritis, 48% had lymphadenopathy, and 24% had jaundice. Regarding flow cytometry results, 26% of the cases were diagnosed with T-ALL and 74% with B-ALL. As for treatment response, 49% of patients experienced relapse. Regarding other laboratory findings, the mean blast cell count was 87.5 ± 11.5. Immunophenotyping revealed the following marker positivity among patients: CD10 (77%), CD34 (71%), CD19 (75%), Human Leukocyte Antigen-DR (51%), CD79a (71%), CD38 (37%), CD13 (12%), CD33 (15%), CD2 (13%), CD7 (21%), CD117 (8%), CD4 (19%), CD11b (7%), CD22 (41%), CD58 (56%), CD81 (44%), TdT (29%), CD3 (26%), and CD8 (15%) (Table 3).

**Table 3:** The clinical presentation of studied cases

	*N* = 100 (%)	Implications
Fever	71(71)	A common symptom of leukemia.
Pallor	98(98)	A significant symptom arising from anemia
Bone Pain	64(64)	Suggests bone marrow infiltration.
Increased Weight	72(72)	Reflects the systemic and local effects of adipose tissue abnormalities
Arthritis	13(13)	Possible Juvenile Idiopathic Arthritis
Lymphadenopathy	48(48)	The infiltration of cancerous lymphoblasts into lymph nodes.
Jaundice	24(24)	May indicate hemolysis
Flow cytometry		
T-ALL	26(26)	Aggressive nature and potential for relapse
B-ALL	74(74)	B-ALL the most common type of ALL in children
Relapsed or finishing treatment Relapsed	49(49)	High rate of relapse; constant monitoring is required.
BLAST (mean ± SD)	87.5 ± 11.5	High blast count consistent with leukemia cells are proliferating
CD10	77(77)	Precursor B cells marker
CD34	71(71)	Hematopoietic stem cells marker
CD19	75(75)	A cell surface marker presents on B cells
Human Leukocyte Antigen-DR	51(51)	Common in early precursor cells.
CD79A	71(71)	Role in B-cell precursor cells
CD38	37(37)	Serves as a potential therapeutic target,
CD13	12(12)	A myeloid marker
CD33	15(15)	A myeloid marker
CD2	13(13)	T-lineage marker.
CD7	21(21)	A cell surface protein
CD117	8(8)	Early hematopoietic progenitor marker.
CD4	19(19)	Helper T-cell marker
CD11b	7(7)	Myeloid lineage
CD22	41(41)	B-lineage marker
CD58	56(56)	role in cell adhesion
CD81	44(44)	Its expression can be a prognostic marker
TdT	29(29)	Lymphoid immaturity marker
CD3	26(26)	T-cell lineage marker
CD8	15(15)	Cytotoxic T-cell marker.

### Comparison of MDR1 (G2677T) rs2032582 genotype between cases and control groups

**Fig. 1:**
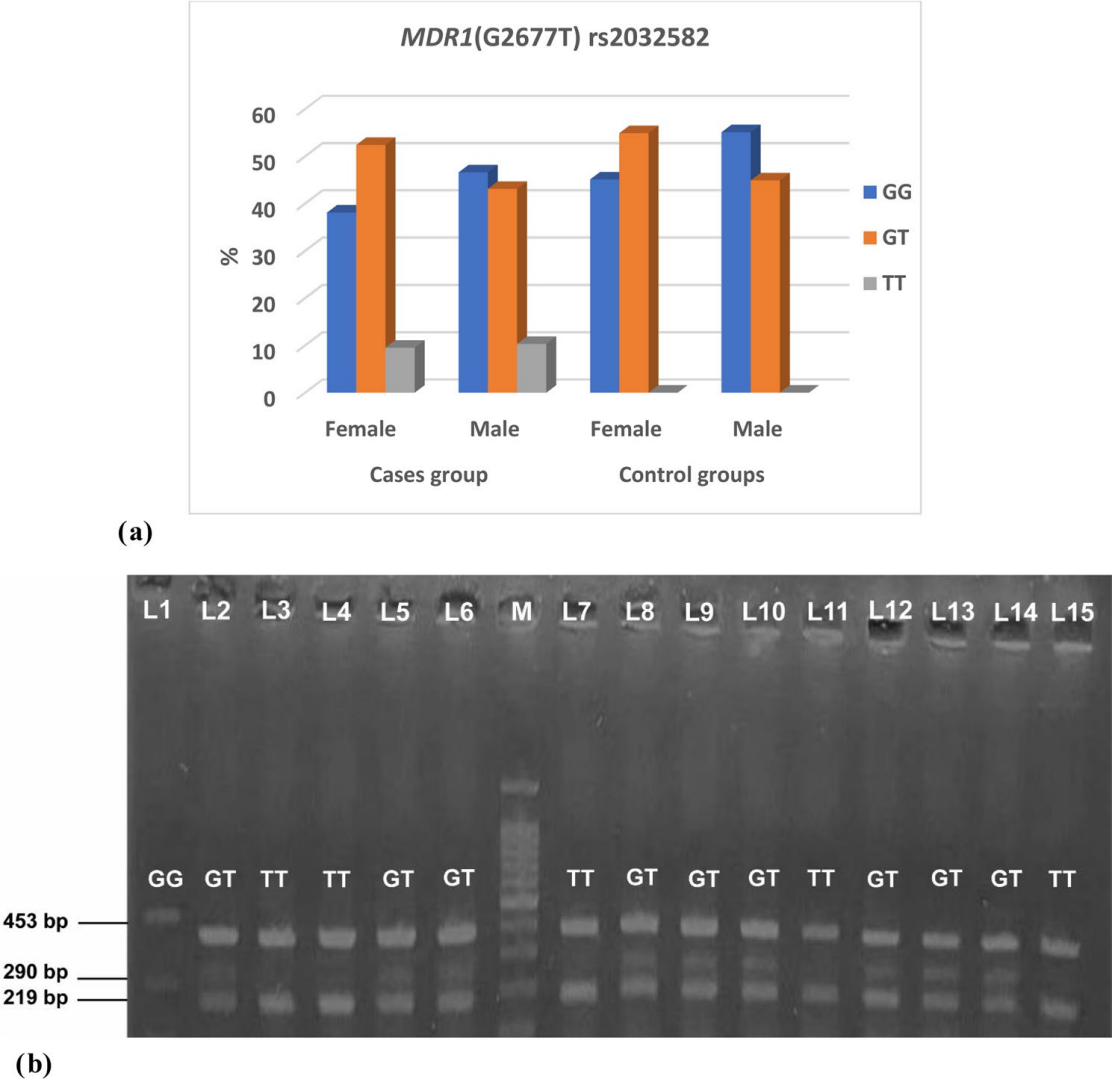
Figure 1 (a) *MDR1* (G2677T) rs2032582 genotype among cases and control groups. b Gel electrophoresis of the T-ARMS-PCR product for the *MDR1* (G2677T) rs2032582 gene variant. Lane 1 showed the homozygous GG genotype with a band at 290 bp and an internal control band at 453 bp. Lanes 3, 4, 7, 11, and 15 showed the homozygous TT genotype with a band at 219 bp and the internal control at 453 bp. Lanes 2, 5, 6, 8-10 and 12-14 represented the heterozygous GT genotype, showing bands at 219 bp (T allele), 290 bp (G allele), and the internal control at 453 bp. M: 100 bp DNA ladder (Thermo Scientific)

The study revealed no significant difference between the case and control groups with the *MDR1* (G2677T) rs2032582 genotype (*p* = 0.051) and allele frequency (*p* = 0.217). Female cases had genotype distribution as follows; 38.1% GG, 52.4% GT and 9.5% TT. male cases had *MDR1* (G2677T) rs2032582 genotype distribution as follows; 46.6% GG, 43.1% GT and 10.3% TT. Female controls had genotype distribution as follows; 45.1% GG and 54.9% GT. Male controls had genotype distribution as follows; 55.1% GG and 44.9% GT (Table 4; Figure 1a, b).

**Table 4:** Comparison of *MDR1* (G2677T) rs2032582 genotype and allele frequencies between case and control groups with HWE analysis

*MDR1* (G2677T) rs2032582	Cases group		Control groups		*p*-value
	Female *N* = 42(%)	Male *N* = 58(%)	Female *N* = 51(%)	Male *N* = 49(%)	
GG	16(38.1)	27(46.6)	23(45.1)	27(55.1)	*P* = 0.051
GT	22(52.4)	25(43.1)	28(54.9)	22(44.9)	
TT	4(9.5)	6(10.3)	0	0	
Allele frequencies	*N* = 84	*N* = 116	*N* = 102	*N* = 98	
G	54	79	74	76	*P* = 0.217
T	30	37	28	22	

Used test: Chi-Square test, data expressed as number (%)

### Distribution of IL18 (607C > A) rs1946518 gene polymorphism in ALL cases compared to controls

**Fig. 2:**
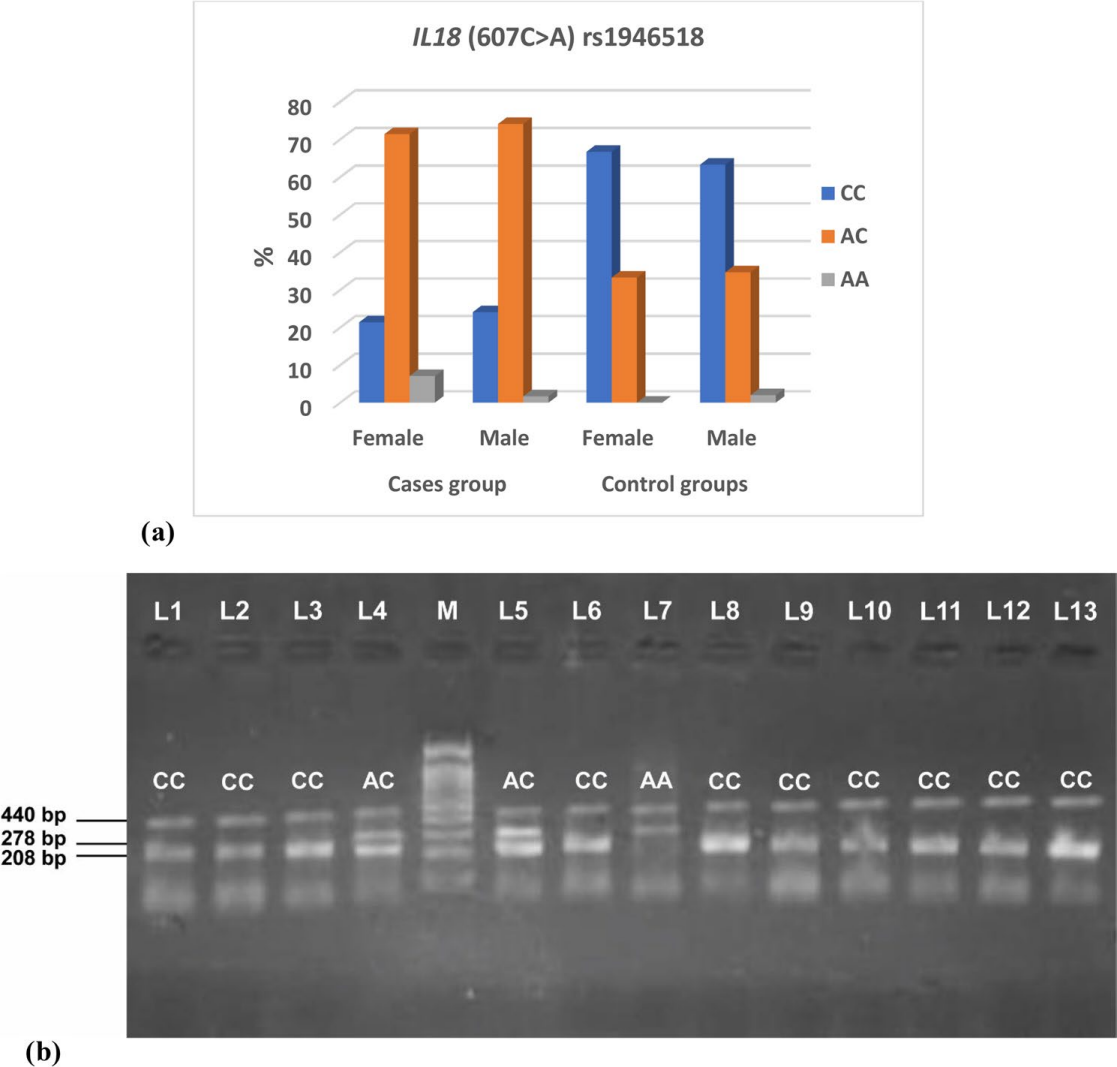
Figure 2 (a) *IL18* (607C >A) rs1946518 genotype among case and control groups. (b) Gel electrophoresis of the T-ARMS-PCR product for *IL18* (607C>A) rs1946518 gene variant. Lane 7 showed the homozygous AA genotype with a band 278 bp and internal control band at 440bp. Lanes 1-3,6 and 8-13 showed the homozygous CC genotype with a band at 208bp and internal control at 440bp. Lanes 4 and 5 showed heterozygous AC genotype at 208bp for C allele, and A allele at 278 bp and internal control at 440bp. M: 100 bp DNA ladder (Thermo scientific)

The study showed a significant difference between the case and control groups with regard to the *IL18* (607C > A) rs1946518 genotype (*p* = 0.001) and allele frequency (*p* = 0.001). Female cases had genotype distribution as follows; 21.4% CC, 71.4% AC and 7.1% AA. male cases had genotype distribution as following; 24.1% CC, 74.1% AC and 1.7% AA. Female controls had genotype distribution as follows; 66.7% CC and 33.3% AC. Male controls had genotype distribution as follows; 63.3% CC, 34.7% AC and 2% AA (Table 5; Figure 2a, b).

**Table 5:** Comparison of *IL18* (607C>A) rs1946518 genotype and allele frequencies between case and control groups with HWE analysis

*IL18* (607C > A) rs1946518	Cases group		Control groups		Test of significance
	Female *N* = 42(%)	Male *N* = 58(%)	Female *N* = 51(%)	Male *N* = 49(%)	
CC	9(21.4)	14(24.1)	34(66.7)	31(63.3)	*P* = 0.001*
AC	30(71.4)	43(74.1)	17(33.3)	17(34.7)	
AA	3(7.1)	1(1.7)	0	1(2.0)	
Allele	*N* = 84	*N* = 116	*N* = 102	*N* = 98	
C	48	71	85	79	*P* = 0.001*
A	36	45	17	19	

Used test: Chi-Square test *statistically significant, data expressed as number (%)

### Association of the IL18 (−137G > C) rs187238 variant with susceptibility to ALL

**Fig. 3:**
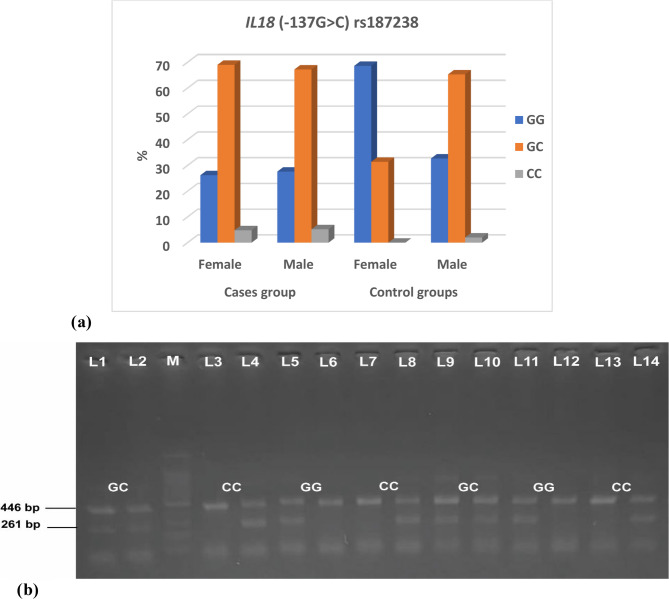
Figure 3 (a) *IL18* (-137G>C) rs187238 genotype among case and control groups. (b) Gel electrophoresis of the T-ARMS-PCR product for *IL18* (-137G>C) rs187238 gene variant. Lanes 1,2 and 9,10 showed heterozygous GC genotype, with both G and C alleles present at 261 bp and internal control band at 446 bp. Lanes 5,6 and 11,12 showed homozygous GG genotype. Lanes 3,4;7,8 and 13,14 showed homozygous CC genotype. M: 100 bp DNA ladder (Thermo scientific)

The analysis revealed a statistically significant difference between the control group and the cases in terms of the *IL18* (−137G > C) rs187238 genotype (*p* = 0.001) and the frequency of the allele (*p* = 0.0006). In female cases, the distribution of the *IL18* (−137G > C) rs187238 genotype was as follows: 26.2% of patients had GG, 69% had GC, and 4.8% had CC. Among male cases, 27.6% had the GG genotype, 67.2% had the GC genotype, and 5.2% had the CC genotype. Female controls were as follows: 68.6% had GG, while 31.4% had GC. Among male controls, 32.7% had the GG genotype, 65.3% had the GC genotype, and 2% had the CC genotype (Table 6; Figure 3a, b).

**Table 6:** Comparison of *IL18* (−137G>C) rs187238 genotype and allele frequencies between case and control groups with HWE analysis

*IL18* (−137G > C) rs187238	Cases group		Control group		*p*-value
	Female *N* = 42(%)	Male *N* = 58(%)	Female *N* = 51(%)	Male *N* = 49(%)	
GG	11(26.2)	16(27.6)	35(68.6)	16(32.7)	*P* = 0.001*
GC	29(69.0)	39(67.2)	16(31.4)	32(65.3)	
CC	2(4.8)	3(5.2)	0	1(2)	
Allele	*N* = 84	*N* = 116	*N* = 102	*N* = 98	
G	51	71	86	64	*P* = 0.0006*
C	33	45	16	34	

Used test: Chi-Square test *statistically significant, data expressed as number (%)

### Genetic polymorphisms of MDR1 (G2677T) rs2032582, IL18 (607C > A) rs1946518, and IL18 (−137G > C) rs187238 among Egyptian children with ALL compared to healthy controls

The comparison between the total cases versus the total control groups includes the following genotypes: A significant difference was detected between the groups regarding the *MDR1* (G2677T) rs2032582 genotype (*p* = 0.005), with a higher frequency of the TT genotype (10% of cases versus 0% of controls, *p* = 0.001). A significant difference was also detected between them regarding the distribution of recessive genes.

A significant difference was detected between studied groups in relation to *IL18* (607C > A) rs1946518 (*p* = 0.001) with a higher frequency of AC genotype among the case than the control group (73% versus 34%, *p* = 0.001). Conversely, the CC genotype was observed at a lower frequency in the case group than in the control group (23% versus 65%, *p* = 0.001). Gene dominance and overdominance also demonstrated significant differences between the groups.

A significant difference was detected between groups concerning *IL18* (−137G > C) rs187238 (*p* = 0.001). The GC genotype was more frequent in the case group compared to the control group (68% versus 48%, *p* = 0.004), whereas the GG genotype was less frequent among the case group than the control group (27% versus 51%, *p* = 0.0005). Gene dominance and overdominance also demonstrated statistically significant difference (Table 7).

**Table 7:** Comparison of studied model genotypes between cases and control

Model genotypes		Cases group	Control groups	Test of significance	*P* value		Odds ratio (95%CI)
		*N* = 100	*N* = 100				
	*MDR1* (G2677T) rs2032582						
Codominante	GG	43(43)	50(50)	*P* = 0.005*		0.320	0.7(0.4–1.3)
	GT	47(47)	50(50)			0.671	0.8(0.5–1.5)
	TT	10(10)	0			0.001*	Undefined
Dominant	GG	43(43)	50(50)	*P* = 0.321			R
	GT + TT	57(57)	50(50)				1.3(0.7–2.3)
Recessive	GG + GT	90(90)	100(100)	*P* = 0.001*			Undefined
	TT	10(10)	0				
Overdominante	GG + TT	53(53)	50(50)	*P* = 0.671			R
	GT	47(47)	50(50)				1.1(0.6–1.9)
	*IL18* (607C > A) rs1946518						
Codominante	CC	23(23)	65(65)	*P* = 0.001*		0.001*	0.1(0.0–0.2.0.2)
	AC	73(73)	34(34)			0.001*	5.2(2.8–9.6)
	AA	4(4)	1(1)			0.174	4.1(0.4–37.5)
Dominant	CC	23(23)	65(65)	*P* = 0.001*			R
	CC + AC	77(77)	35(35)				6.2(3.3–11.5)
Recessive	CC + AC	96(96)	99(99)	*P* = 0.170			R
	AA	4(4)	1(1)				4.1(0.4–37.5)
Overdominante	CC + AA	27(27)	66(66)	*P* = 0.001*			R
	AC	73(73)	34(34)				2.2(2.8–9.6)
	*IL18* (−137G > C) rs187238						
Codominante	GG	27(27)	51(51)	*P* = 0.001*		0.0005*	0.3(0.1–0.6)
	GC	68(68)	48(48)			0.004*	2.3(1.2–4.0.2.0)
	CC	5(5)	1(1)			0.097	5.2(0.5–45.4)
Dominant	GG	27(27)	51(51)	*P* = 0.0005*			R
	CC + CC	73(73)	49(49)				2.8(1.5–5.0.5.0)
Recessive	GG + GC	95(95)	99(99)	*P* = 0.097			5.2(0.5–45.4)
	CC	5(5)	1(1)				R
Overdominante	GG + CC	32(32)	52(52)	*P* = 0.004*			R
	GC	68(68)	48(48)				2.3(1.2–4.0.2.0)
	AG	60(60)	88(88)				R

Used test: Chi-Square test *statistically significant, data expressed as number (%). 95% CI: 95% confidence interval for the difference between the means for both groups

### Relation between MDR1 (G2677T) rs2032582, IL18 (607C > A) rs1946518, IL18 (−137G > C) rs187238 and clinical, laboratory findings of the cases

This study demonstrated significant relation between *MDR1* (G2677T) rs2032582 genotype and CD117 (*p* = 0.007) with higher frequency of CD117 expression among GT genotype (17%). Additionally, a significant relation between *IL18* (607C > A) rs1946518 genotype and bilirubin (*p* = 0.049) with higher median bilirubin among AC genotype followed by CC and the least for AA. Median platelet count was higher among AA genotype followed by CC and the least for AC genotype with statistically significant relation platelet count (*p* = 0.038). Moreover, a significant association between the *IL18* (−137G > C) rs187238 genotype and the presence of bone pain (*p* = 0.002), with a higher frequency of bone affection observed in cases with the GG genotype (81.5%), followed by the GC genotype (61.8%). A statistically significant association was also found with CD58 (*p* = 0.030) (Table 8).

**Table 8:** Comparison of *MDR1* (G2677T) rs2032582, *IL18* (607C>A) rs1946518, *IL18* (−137G>C) rs187238 genotypes and clinical, laboratory findings of studied cases

Variable	Genotype	*P* value
*MDR1* (G2677T) rs2032582		
CD117	GG: 0 (0%) GT: 8 (17%) TT: 0 (0%)	0.007*
*IL18* (607C > A) rs1946518		
Bilirubin	CC: 0.7 (0.2–3) AC: 0.7 (0.2–4.3) AA: 0.4 (0.3–0.6)	0.049*
Platelet count	CC: 242 (18–601) AC: 174 (11–556) AA: 333 (1375–400)	0.038*
*IL18* (−137G > C) rs187238		
Bone pain	GG: 22 (81.5%) GC: 42 (61.8%) CC: 0 (0%)	0.002*
CD58	GG: 20 (74.1%) GC: 32 (47.1%) CC: 4 (80%)	0.030*

Used tests: Chi-Square test, One Way ANOVA test, Kruskal Wallis test *statistically significant, data expressed as number (%), median (range).

## Discussion

One of the most important *MDR1* gene polymorphisms is the G2677T SNP. This polymorphism has been significantly associated with variations in plasma concentrations of P-gp substrates, impacting drug absorption and efficacy [27]. To be more specific, individuals who have the G2677T variation may have either higher or decreased levels of these substrates in their plasma, which can have an impact on the treatment responses and drug resistance that occur in cancer therapy [28]. Variants in the *MDR1* gene, such as the G2677T polymorphism (rs2032582), have been investigated to determine the extent to which they operate as a risk factor for leukemia in Egyptian children.

The distribution of genotypes and no significant differences between cases and controls are the main concerns of this study. Comparing all cases with the control group, no significant difference in the distribution of the genotypes and allele frequencies could be shown for *MDR1*(G2677T) rs2032582 genotype (*p* = 0.051) and allele frequency (*p* = 0.217). Therefore, the *MDR1* (G2677T) SNP may be a weak predictor of disease with consideration to all patients. The findings of this investigation demonstrated a strong relationship of the TT genotype. The large discrepancy in the frequency of the TT genotype indicates a recessive nature, this variant may confer the risk of a disease. Therefore, further research is needed to elucidate the effects of the *MDR1* (G2677T) rs2032582 genotype in other populations and its potential role in the development of the disease and the strategies used for treatment. This is consistent with the results of Ammar et al. [29] in Tunisia, which show a significant difference in the distribution of *MDR1* G2677T genotype between cancer patients and healthy individuals. A higher frequency of the TT genotype in patients with leukemia was noted when compared with controls, and the patent association of TT genotype with disease susceptibility is indicated.

Among Serbian patients with ulcerative colitis, carriers of the T allele of the G2677T SNP in the *MDR1* gene are more frequent in comparison to healthy controls based on the study [30]. The findings of this study suggest the role of the T allele and susceptibility to ulcerative colitis in the population. This evidence tends to confirm that these polymorphisms have an etiologic predisposing role in inflammatory bowel disease. Consistent with the report of Fu et al. [31], which assessed the association of *MDR1* gene polymorphisms with cardiovascular diseases, there was no significant association between the G2677T polymorphism and susceptibility to the disease; it seems that the risk of this polymorphism in the occurrence of cardiovascular diseases in these people is not considerable. According to the findings of the research carried out by Yan et al. [32], which conducted a meta-analysis of the association between the *MDR1* G2677T polymorphism and the risk of leukemia, the investigation did not uncover any significant association in the general population. More specifically, the results indicated that the TT genotype did not show a statistically significant increase in leukemia risk compared to the TG and GG genotypes across different analytical models.

Our findings for the *MDR1* (G2677T) rs2032582 gene show significant associations with disease susceptibility in both female and male patients, particularly for the TT genotype. While many studies support these findings, some contradict them, highlighting the complexity of genetic influences on health. Further research is needed to explore the nuances of these associations and their implications for clinical practice. Our study also shows a statistically significant association between the *MDR1* (G2677T) rs2032582 genotype and CD117 (*p* = 0.007) a tyrosine kinase receptor involved in cell signaling and hematopoiesis with a higher frequency of CD117 expression among the GT genotype (17%). This finding helps us understand functional gene interactions in cell biology, especially in hematopoiesis and related disorders such as acute leukemia in children.

The *IL18* cytokine is integral to the immune response, which plays a critical role in activating immune cells, particularly T cells and natural killer cells [33]. Genetic variations in *IL18* can lead to altered immune responses, potentially impacting the development of lymphoid malignancies such as ALL [34]. Insufficient immune surveillance, stemming from genetic predispositions, may facilitate the proliferation of malignant cells [35]. The present study of the *IL18* (607C >A) rs1946518 genotype distribution among case and control groups reveals significant differences. A statistically significant higher frequency of the A allele (either the heterozygous AC or homozygous AA form) may have an increased susceptibility to the disease among cases compared to controls, indicating a potential risk factor associated with the A allele. The AA genotype, although relatively rare, appeared more frequently in female cases (7.1%) than in any control group. A significant difference was noted in dominant gene distribution (CC + AC) and overdominance gene distribution (AC), with higher percentages in the case groups compared to controls.

The AC genotype may result in altered *IL18* expression or receptor signaling, which can impact immune responses and potentially play a role in the development of disease [36]. The frequency of the CC genotype was notably greater in the control groups than in the case groups (*p* = 0.001). This observation suggests that individuals possessing this genotype might have an inherent protective advantage against diseases associated with immune responses. Confirming the results of our study is the study conducted by Al-ardawy et al. [37] as their study demonstrated the presence of statistically significant differences in the genetic and allelic frequencies of *IL18* rs1946518, which are associated with the likelihood of developing ulcerative colitis. The current findings, supported by the study of Yang et al. [38], show that the meta-analysis results indicate that the *IL18* gene promoter − 607C >A polymorphism is significantly associated with an increased risk of cancer in general, particularly in cases of nasopharyngeal cancer and gastrointestinal cancer.

The data indicate that the *IL18* (−137G >C) variation may contribute to an elevated risk of pediatric leukemia development. The observed increased prevalence of the GC genotype in patients relative to controls suggests that this genotype may heighten the risk of leukemia, as shown by an odds ratio, whereas the frequency of the GG genotype was significantly higher in the control groups than in the case groups. According to Chen et al. [39], the statistical rise in the prevalence of the C allele among cases (either in the heterozygous GC or homozygous CC) further substantiates the concept that this allele may contribute to the etiology of juvenile leukemia. The presence of the C allele may be associated with altered *IL18* expression, which is crucial for immune regulation and inflammation, both of which are vital in cancer progression. Regarding dominant and overdominant gene distribution, 51% of the control group exhibit GG genotypes, in contrast to 27% in the case group (odds ratio = 0.355). This finding indicates that possessing the GG genotype as a preventive agent reduces the risk of leukemia in patients. The overdominance effect is further highlighted by the 68% prevalence of the GC genotype, which substantially increases the risk of pediatric leukemia, reinforcing the notion of heightened susceptibility linked to this genotype to be infected with malignant diseases [40].

The results of the study conducted by Lau et al. [41] revealed that the *IL18* (−137G >C) polymorphism is strongly correlated with an elevated risk of hepatocellular carcinoma, but the (607C >A) rs1946518 polymorphism did not show a significant association with hepatocellular carcinoma susceptibility. The data indicate that *IL18* polymorphisms do not consistently influence cancer risk. The study’s results indicate significant disparities in the distribution of the *IL18* (−137G >C) rs187238 genotypes between children with leukemia and the control group. There is a notable disparity in genotype distributions between these two populations. Gene dominance and overdominance both exhibit a statistically significant difference.

These findings suggest that the *IL18* polymorphism may influence bilirubin metabolism and play a role in platelet production. That means it may have a role in several blood diseases and in how the body reacts to inflammation. The results of our study revealed the presence of a significant association between the *IL18* (607C >A) rs1946518 and bilirubin (*p* = 0.049). Median bilirubin was highest in the AC genotype, followed by the CC genotype, and was lowest in the AA genotype. Median platelet count was higher in AA than in CC and AC genotypes with a statistically significant association (*p* = 0.038). Such findings suggest that the *IL18* polymorphism might be involved in the catabolism of bilirubin and in the genesis of platelets. Meaning it might be involved in a variety of blood disorders and the body’s response to inflammation [42].

In contrast, the distribution of the *1L18* (−137G >C) rs187238 genotype and allele frequency did not differ in males. This suggests a lack of association between *IL18* and leukemia, possibly indicating that *IL18* demonstrates a more pronounced genetic influence in females than males. This may be because males and females have different hormones or immunological responses. The *IL18* (−137G >C) polymorphism is associated with several diseases, particularly in Asian populations. This genetic variant is linked to an increased risk of nasopharyngeal carcinoma. Studies on head and neck cancer (HNC) have shown a connection between *IL18* genetic variation and a predisposition to developing cancer [43]. The *IL18* (−137G >C) polymorphism is also associated with an increased risk of developing type 1 diabetes in the Egyptian population. This finding suggests that variations in the *IL18* gene may be critical in the development of autoimmune diseases such as type 1 diabetes [44]. This study found that there is a correlation between the *IL18* (−137G >C) rs187238 genotype and the number of bone lesions, and that this correlation is statistically significant (*p* = 0.002). There was a significantly higher incidence of bone lesions among those with the GG genotype compared to those with the GC genotype (81.5% and 61.8%, respectively).

## Conclusions

This study highlights the potential genetic influence of *IL18* and *MDR1* polymorphisms on pediatric leukemia susceptibility. While the *MDR1* (G2677T) rs2032582 genotype showed no statistically significant association with disease risk, a possible weak predictive value cannot be entirely ruled out. In contrast, significant associations were observed for *IL18* gene variants. The *IL18* (607C > A) rs1946518 A allele—particularly in AC and AA genotypes—was more prevalent among cases, suggesting an increased susceptibility to leukemia. Furthermore, the *IL18* (−137G > C) GC genotype demonstrated a strong association with heightened disease risk, whereas the GG genotype appeared to offer a protective effect. These findings support the role of *IL18* gene variations as potential genetic markers for increased pediatric leukemia risk.

## Supplementary Information

Supplementary Material 1

## Data Availability

All the data analyzed during the current study are available from the corresponding author on reasonable request (Mai M. Madkour).

## Abbreviations

ALL: Acute Lymphoblastic Leukemia
MDR1: Multidrug Resistance Gene
P-gp: P-glycoprotein
IL18: Interleukin 18
TLC: Total Leukocyte Count
HB: Hemoglobin
GPT: Glutamate Pyruvate Transaminase
GOT: Glutamate Oxaloacetate Transaminase
T-ARMS-PCR: Tetra-Primer Amplification Refractory Mutation System-Polymerase Chain Reaction
HWE: Hardy-Weinberg Equilibrium
SNP: Single Nucleotide Polymorphism
