# Supplementary material for: Genetic insights into acute lymphoblastic leukemia: the role of MDR1 and IL18 polymorphisms in Egyptian children
Source: BMC Cancer. 2025 Nov 19;25:1792. doi: 10.1186/s12885-025-15132-6 (PMC12628906; doi:10.1186/s12885-025-15132-6)
Supplement: Supplementary file 1 — Supplementary Material 1 [file 12885_2025_15132_MOESM1_ESM.docx]

**Table (1):** Comparison of *MDR1* (G2677T) rs2032582 genotypes with clinical and laboratory findings in studied cases

| Variable | *MDR1* (G2677T) rs2032582 | | | P value |
| --- | --- | --- | --- | --- |
|  | **GG**  **N=43** | **GT**  **N=47** | **TT**  **N=10** |  |
| Fever | 32(74.4) | 30(63.8) | 9(90) | 0.205 |
| Pallor | 42(97.7) | 46(97.9) | 10(100) | 0.891 |
| Bone pain | 26(60.5) | 32(68.1) | 6(60) | 0.725 |
| Weight | 32(74.4) | 32(68.1) | 8(80) | 0.670 |
| Arthritis | 5(11.6) | 7(14.9) | 1(10) | 0.861 |
| Lymphadenopathy | 22(51.2) | 19(40.4) | 7(70) | 0.203 |
| Jaundice | 11(25.6) | 11(23.4) | 2(20) | 0.925 |
| Flow cytometry  T-ALL  B-ALL | 9(20.9)  34(79.1) | 15(31.9)  32(68.1) | 2(20)  8(80) | 0.446 |
| CD10 | 36(83.7) | 34(72.3) | 7(70) | 0.377 |
| CD34 | 33(76.7) | 30(63.8) | 8(80) | 0.324 |
| CD19 | 34(79.1) | 33(70.2) | 8(80) | 0.581 |
| Human Leukocyte Antigen-DR | 21(48.8) | 24(51.1) | 6(60) | 0.817 |
| CD79A | 32(74.4) | 32(68.1) | 7(70) | 0.801 |
| CD38 | 13(30.2) | 19(40.4) | 5(50) | 0.405 |
| CD13 | 3(7) | 8(17) | 1(10) | 0.335 |
| CD33 | 7(16.3) | 6(12.8) | 2(20) | 0.804 |
| CD2 | 9(20.9) | 3(6.4) | 1(10) | 0.117 |
| CD7 | 8(18.6) | 12(25.5) | 1(10) | 0.482 |
| CD117 | 0 | 8(17) | 0 | 0.007* |
| CD4 | 9(20.9) | 10(21.3) | 0 | 0.271 |
| CD11b | 3(7) | 4(8.5) | 0 | 0.632 |
| CD22 | 21(48.8) | 16(34) | 4(40) | 0.361 |
| CD58 | 25(58.1) | 28(59.6) | 3(30) | 0.216 |
| CD81 | 17(39.5) | 23(48.9) | 4(40) | 0.645 |
| TdT | 15(34.9) | 13(27.7) | 1(10) | 0.284 |
| CD3 | 8(18.6) | 16(34) | 2(20) | 0.224 |
| CD8 | 6(14) | 8(17) | 1(10) | 0.825 |
| TLC | 4.38±3.29 | 4.082±2.51 | 4.44±2.97 | 0.868 |
| HB | 9.44±1.52 | 9.42±1.89 | 8.65±2.26 | 0.425 |
| Albumin | 4.27±0.45 | 4.18±0.59 | 3.94±0.56 | 0.279 |
| GPT | 62(11-318) | 51(9-794) | 53.12(15-231) | 0.639 |
| GOT | 31(10-303) | 37(15-561) | 26.15(20.52-172) | 0.269 |
| Creatinine | 0.5(0.2-1.2) | 0.5(0.3-51.3) | 0.55(0.2-0.9) | 0.247 |
| Bilirubin | 0.7(0.2-2.4) | 0.7(0.3-4.39) | 0.60(0.2-3.7) | 0.906 |
| RBC | 3.09(1.96-4.84) | 3.39(1.61-4.37) | 2.65(1.38-4.67) | 0.496 |
| Platelet count | 175(11.8-601) | 190(18-504) | 105.11(24.5-249) | 0.062 |
| ALP | 126(60-607.2) | 129(59-322) | 122.5(42-282) | 0.877 |
| Na^+^ | 144(130-227) | 143(122-218) | 134(122-158) | 0.132 |
| K | 3.2(1.8-5.5) | 3(1-8.2) | 3.05(2.3-5.1) | 0.778 |
| CA | 0.54(0.25-0.93) | 0.445(0.10-0.97) | 0.535(0.43-.85) | 0.153 |
| LDH | 231(156-859) | 238(107-622) | 217(159-2081) | 0.398 |
| Lymph | 8(2-36) | 9(1-27) | 7.5(3-55) | 0.939 |
| Blast | 2(1-87) | 2(1-97) | 2(1-5) | 0.470 |

Used tests: Chi-Square test, One Way ANOVA test, Kruskal Wallis test *statistically significant, data expressed as number (%), mean±SD, median (range).

**Table (2):** Comparison of *IL18* (607C>A) rs1946518 genotypes with clinical and laboratory findings in studied cases

| **Variable** | ***IL18* (607C>A) rs1946518** | | | **P value** |
| --- | --- | --- | --- | --- |
|  | **CC**  **N=23** | **AC**  **N=73** | **AA**  **N=4** |  |
| **Fever** | 18(78.3) | 51(69.9) | 2(50) | 0.474 |
| **Pallor** | 22(95.7) | 72(98.6) | 4(100) | 0.645 |
| **Bone pain** | 10(43.5) | 51(69.9) | 3(75) | 0.064 |
| **Weight** | 16(69.6) | 52(71.2) | 4(100) | 0.439 |
| **Arthritis** | 2(8.7) | 10(13.7) | 1(25) | 0.632 |
| **Lymphadenopathy** | 11(47.8) | 34(46.6) | 3(75) | 0.541 |
| **Jaundice** | 7(30.4) | 17(23.3) | 0 | 0.405 |
| **Flow cytometry**  **T-ALL**  **B-ALL** | 7(30.4)  16(69.6) | 18(24.7)  55(75.3) | 1(25)  3(75) | 0.858 |
| **CD10** | 18(78.3) | 56(76.7) | 3(75) | 0.984 |
| **CD34** | 15(65.2) | 52(71.2) | 4(100) | 0.366 |
| **CD19** | 17(73.9) | 55(75.3) | 3(75) | 0.991 |
| **Human Leukocyte Antigen-DR** | 11(47.8) | 37(50.7) | 3(75) | 0.601 |
| **CD79A** | 16(69.6) | 53(72.6) | 2(50) | 0.615 |
| **CD38** | 10(43.5) | 27(37) | 0 | 0.251 |
| **CD13** | 2(8.7) | 9(12.3) | 1(25) | 0.642 |
| **CD33** | 3(13) | 11(15.1) | 1(25) | 0.826 |
| **CD2** | 3(13) | 9(12.3) | 1(25) | 0.764 |
| **CD7** | 5(21.7) | 15(20.5) | 1(25) | 0.973 |
| **CD117** | 1(4.3) | 7(9.6) | 0 | 0.602 |
| **CD4** | 7(30.4) | 11(15.1) | 1(25) | 0.249 |
| **CD11b** | 2(8.7) | 5(6.8) | 0 | 0.817 |
| **CD22** | 13(56.5) | 26(35.6) | 2(50) | 0.192 |
| **CD58** | 12(52.2 | 42(57.5) | 2(50) | 0.876 |
| **CD81** | 9(39.1) | 34(46.6) | 1(25) | 0.605 |
| **TdT** | 8(34.8) | 20(27.4) | 1(25) | 0.780 |
| **CD3** | 7(30.4) | 18(24.7) | 1(25) | 0.858 |
| **CD8** | 5(21.7) | 9(12.3) | 1(25) | 0.463 |
| **TLC** | 4.48±2.89 | 4.09±2.86 | 5.81±3.69 | 0.471 |
| **HB** | 9.58±1.28 | 9.27±1.91 | 9.49±1.95 | 0.756 |
| **Albumin** | 4.26±0.45 | 4.15±0.56 | 4.31±0.58 | 0.601 |
| **GPT** | 53.5(9-318) | 54(11-794) | 55.5(17-256) | 0.960 |
| **GOT** | 30.19(18-303) | 35(10-561) | 33.5(26.11-45) | 0.516 |
| **Creatinine** | 0.40(0.2-0.6) | 0.5(0.2-51.3) | 0.45(0.4-0.7) | 0.092 |
| **Bilirubin** | 0.7(0.2-3) | 0.7(0.2-4.39) | 0.4(0.3-0.6) | 0.049* |
| **RBC** | 3.09(1.96-4.67) | 3.07(1.38-4.84) | 3.49(2.17-4.22) | 0.882 |
| **Platelet count** | 242(18-601) | 174(11.8-556) | 333(1375-400.1) | 0.038* |
| **ALP** | 126(78-232) | 131(42-607.2) | 135(60-214) | 0.967 |
| **Na^+^** | 142(126-227) | 143(122-218) | 150(135-161) | 0.737 |
| **K** | 3.5(1.1-4.9) | 3.1(1.4-8.2) | 3(1-3) | 0.425 |
| **CA** | 0.53(0.1-0.87) | 0.52(0.1-0.97) | 0.605(0.1-0.85) | 0.955 |
| **LDH** | 231(107-418) | 232(150-2081) | 256.5(217-283) | 0.419 |
| **Lymph** | 6(2-36) | 9(1-55) | 10(7-12) | 0.223 |
| **Blast** | 2(1-21) | 2(1-21) | 2(1-5) | 0.645 |

Used tests: Chi-Square test, One Way ANOVA test, Kruskal Wallis test *statistically significant, data expressed as number (%), mean±SD, median (range).

**Table (3):** Comparison of *IL18* (-137G>C) rs187238 genotypes with clinical and laboratory findings in studied cases

| **Variable** | ***IL18* (-137G>C) rs187238** | | | **P value** |
| --- | --- | --- | --- | --- |
|  | **GG**  **N=27** | **GC**  **N=68** | **CC**  **N=5** |  |
| **Fever** | 17(63) | 51(75) | 3(60) | 0.434 |
| **Pallor** | 26(96.3) | 67(98.5) | 5(100) | 0.741 |
| **Bone pain** | 22(81.5) | 42(61.8) | 0 | 0.002* |
| **Weight** | 20(74.1) | 50(73.5) | 2(40.0) | 0.262 |
| **Arthritis** | 3(11.1) | 10(14.7) | 0 | 0.604 |
| **Lymphadenopathy** | 11(40.7) | 36(52.9) | 1(20) | 0.246 |
| **Jaundice** | 7(25.9) | 17(75) | 0 | 0.434 |
| **Flow cytometry**  **T-ALL**  **B-ALL** | 10(37)  17(63) | 16(23.5)  52(76.5) | 0  5(100) | 0.159 |
| **CD10** | 17(63) | 56(82.4) | 4(80) | 0.127 |
| **CD34** | 17(63) | 50(73.5) | 4(80) | 0.534 |
| **CD19** | 17(63) | 53(77.9) | 5(100) | 0.131 |
| **Human Leukocyte Antigen-DR** | 14(51.9) | 36(52.9) | 1(20) | 0.362 |
| **CD79A** | 15(55.6) | 53(77.9) | 3(60) | 0.082 |
| **CD38** | 8(29.6) | 27(39.7) | 2(40) | 0.650 |
| **CD13** | 1(3.7) | 11(16.2) | 0 | 0.168 |
| **CD33** | 4(14.8) | 10(14.7) | 1(20) | 0.950 |
| **CD2** | 3(11.1) | 9(13.2) | 1(20) | 0.859 |
| **CD7** | 9(33.3) | 11(16.2) | 1(20) | 0.180 |
| **CD117** | 2(7.4) | 6(8.8) | 0 | 0.775 |
| **CD4** | 9(33.3) | 10(14.7) | 0 | 0.061 |
| **CD11b** | 2(7.4) | 5(7.4) | 0 | 0.820 |
| **CD22** | 8(29.6) | 30(44.1) | 3(60) | 0.292 |
| **CD58** | 20(74.1) | 32(47.1) | 4(80) | 0.030* |
| **CD81** | 14(51.9) | 29(42.6) | 1(20) | 0.388 |
| **TdT** | 9(33.3) | 20(29.4) | 0 | 0.318 |
| **CD3** | 11(40.7) | 15(22.1) | 0 | 0.069 |
| **CD8** | 7(25.9) | 8(11.8) | 0 | 0.137 |
| **TLC** | 4.33±2.96 | 4.17±2.92 | 4.77±2.40 | 0.894 |
| **HB** | 9.62±1.83 | 9.21±1.78 | 9.71±1.36 | 0.543 |
| **Albumin** | 4.23±0.49 | 4.15±0.56 | 4.41±0.20 | 0.503 |
| **GPT** | 51(11-381) | 54(9-794) | 53.5(17-67) | 0.780 |
| **GOT** | 37(15-275) | 32.5(10-561) | 21.7(16-50) | 0.141 |
| **Creatinine** | 0.5(0.4-51.3) | 0.5(0.2-1.2) | 0.6(0.4-0.8) | 0.790 |
| **Bilirubin** | 0.6(0.2-4.28) | 0.7(0.2-4.39) | 0.68(0.4-0.8) | 0.728 |
| **RBC** | 3.08(2.13-4.54) | 3.08(1.38-4.84) | 3.39(2.76-4.67) | 0.636 |
| **Platelet count** | 187(18-504) | 175.5(11.8-601) | 155(101-369) | 0.979 |
| **ALP** | 112(59-607.2) | 137.5(42-322) | 78(65-192) | 0.133 |
| **Na^+^** | 142(129-202) | 144(122-227) | 144(126-146) | 0.866 |
| **K** | 3.3(1.0-7.1) | 3.0(1.1-8.2) | 3.1(2.4-4.9) | 0.407 |
| **CA** | 0.5(0.1-0.97) | 0.525(0.1-0.97) | 0.44(0.36-0.85) | 0.866 |
| **LDH** | 239(107-2081) | 227(156-859) | 243(169-328) | 0.409 |
| **Lymph** | 8(1-27) | 9(2-55) | 9(3-13) | 0.944 |
| **Blast** | 2(1-97) | 2(1-87) | 1(1-2) | 0.228 |

Used tests: Chi-Square test, One Way ANOVA test, Kruskal Wallis test *statistically significant, data expressed as number (%), mean ± SD, median (range).
